# Two's Company: A Case Report Presenting Diagnostic and Surgical Challenges of a Rare Congenital Duplicated Gallbladder

**DOI:** 10.7759/cureus.85241

**Published:** 2025-06-02

**Authors:** Joshua D Batista, Andrew Liepshutz, Brooke A Merdjane, Nima Khosravani, Jorge Rabaza

**Affiliations:** 1 General Surgery, Baptist Hospital of Miami, Miami, USA; 2 General Surgery, Florida International University, Herbert Wertheim College of Medicine, Miami, USA

**Keywords:** cholecystectomy, cholecystitis, duplicated, duplicated gallbladder, gallbladder, robotic, twin

## Abstract

Duplicated gallbladders are a rare congenital anomaly. Most cases are asymptomatic, but duplicated gallbladders can present significant diagnostic and surgical challenges, particularly due to the risk of bile duct injury or retained stones if not recognized preoperatively. Advanced imaging modalities such as ultrasonography, magnetic resonance cholangiopancreatography, and intraoperative cholangiography are often required for accurate diagnosis, though variability in anatomical presentation complicates preoperative identification. Clinical vigilance is therefore essential, especially in atypical biliary presentations. We present the case of a 56-year-old male patient with a duplicated gallbladder who successfully underwent robotic-assisted cholecystectomy. The case report adheres to the CARE (CAse REport) guidelines to ensure transparency and completeness, detailing the clinical history, diagnostic approach, surgical management, and outcomes.

## Introduction

Duplicated gallbladders (DG) represent an exceedingly rare congenital anomaly, with an estimated prevalence of one in 3800 to one in 4000 individuals undergoing cholecystectomy [1]. This condition arises during the fifth or sixth week of embryonic development due to incomplete division of the cystic bud. Most cases are asymptomatic and discovered incidentally during imaging or surgery, but DG poses significant diagnostic and surgical challenges, increasing the risk of complications such as bile duct or artery injury and retained stones if not recognized preoperatively [1,2]. Accurate diagnosis often requires imaging modalities like ultrasonography, magnetic resonance cholangiopancreatography (MRCP), and intraoperative cholangiography, but its rarity and variable anatomy make preoperative identification difficult, emphasizing the need for clinical vigilance in atypical biliary presentations. Here we discuss our meticulous approach to a duplicated gallbladder in the case of a 56-year-old male patient with mild cholecystitis who underwent surgery and had a full recovery.

## Case presentation

**Figure 1:**
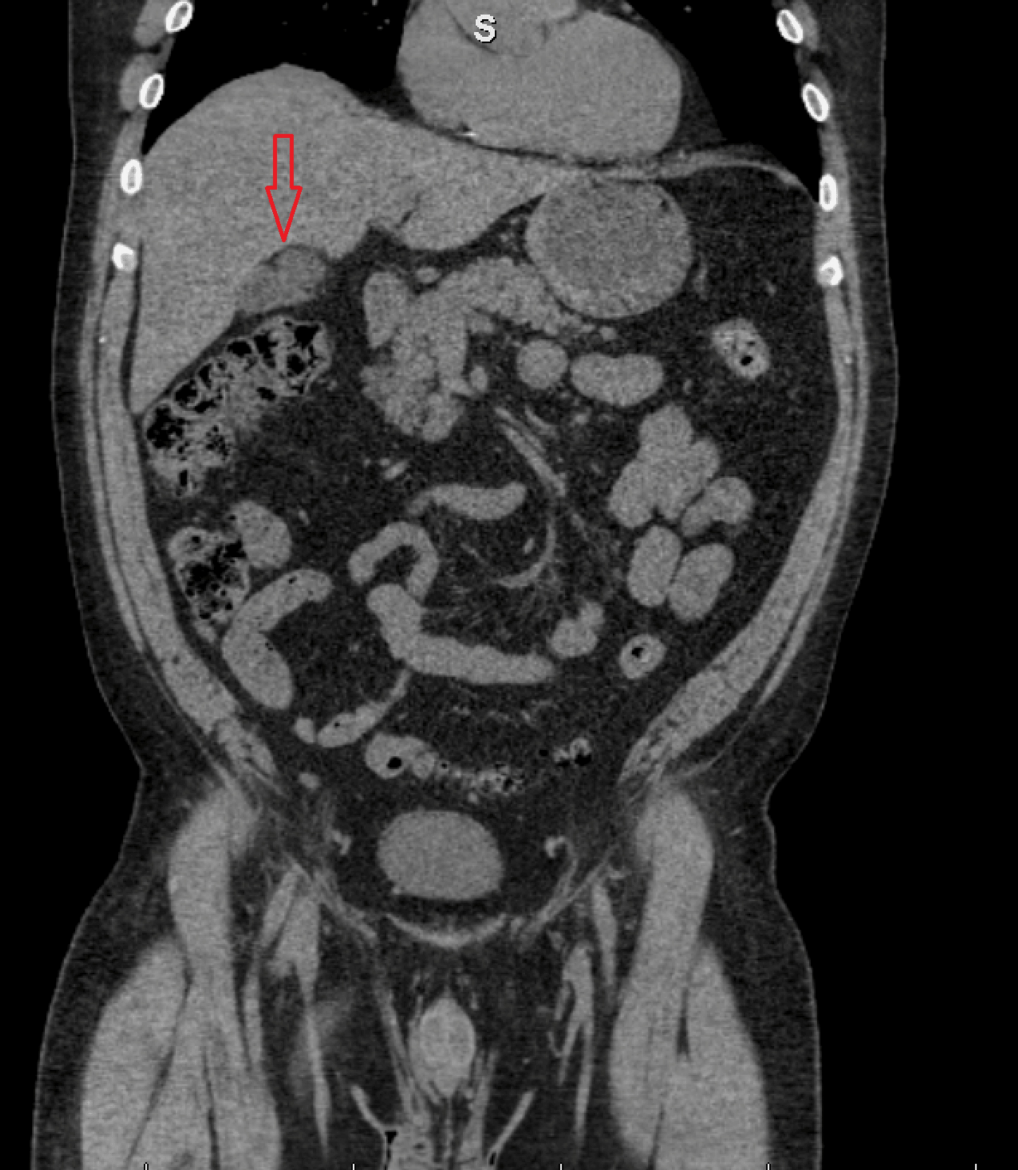
Abdominal pelvic CT without contrast showing gallbladder wall thickening.

A 56-year-old male patient with a history of hypertension, psoriatic arthritis, and morbid obesity presented to the emergency room with significant right upper quadrant pain associated with nausea and vomiting that started that evening. Similar episodes had occurred for the last two weeks. Physical exam was unremarkable as well as vitals, complete blood count (CBC), and complete metabolic panel (CMP). An abdominal pelvic computed tomography (CT) (Figure 1) without contrast demonstrated concern for mild gallbladder wall thickening suggestive of cholecystitis.

**Figure 2:**
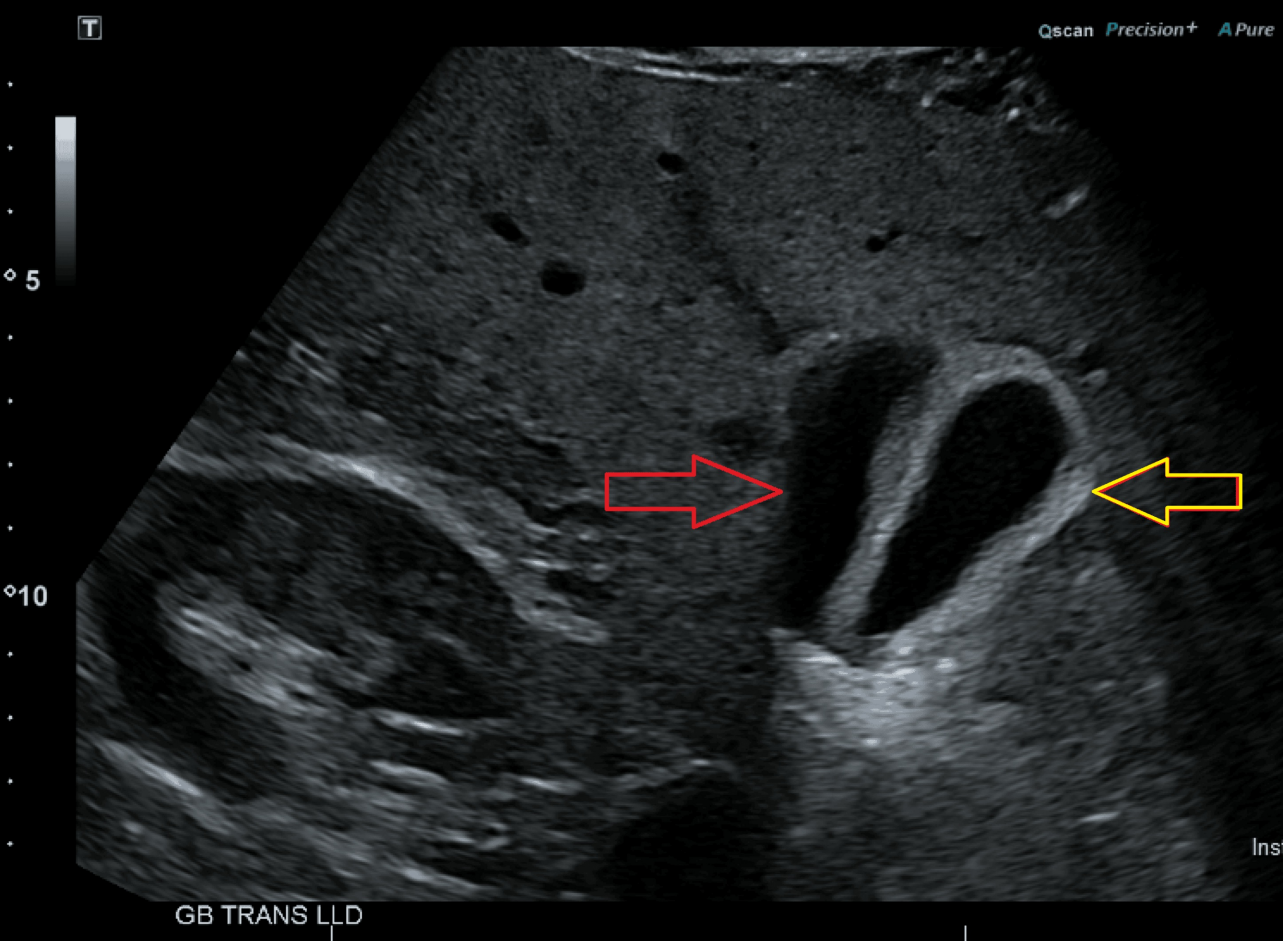
RUQ ultrasound showing duplicated gallbladder. Yellow arrow depicts a thickened gallbladder wall. Red arrow depicts a duplicated gallbladder. RUQ: right upper quadrant.

A routine right upper quadrant (RUQ) ultrasound (Figure 2) examination then demonstrated no calcified stones; however, there was wall thickening (6 mm), and concerns for a duplicated gallbladder.

**Figure 3:**
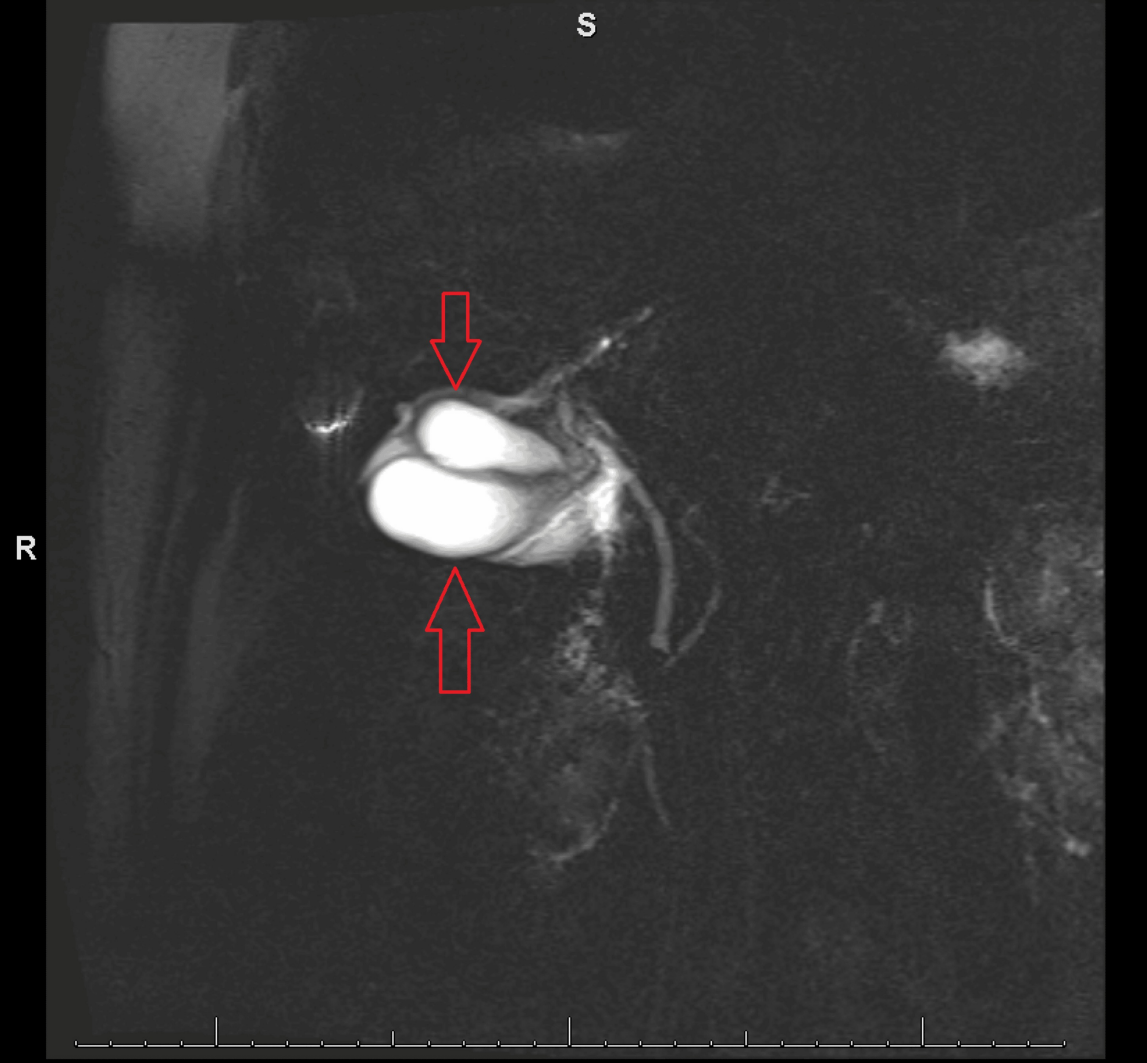
MRCP confirmed duplicated gallbladder. MRCP: magnetic resonance cholangiopancreatography.

An MRCP (Figure 3) was then performed to better evaluate the aberrant anatomy, which confirmed a duplicated gallbladder containing sludge and diffuse wall edema.

**Figure 4:**
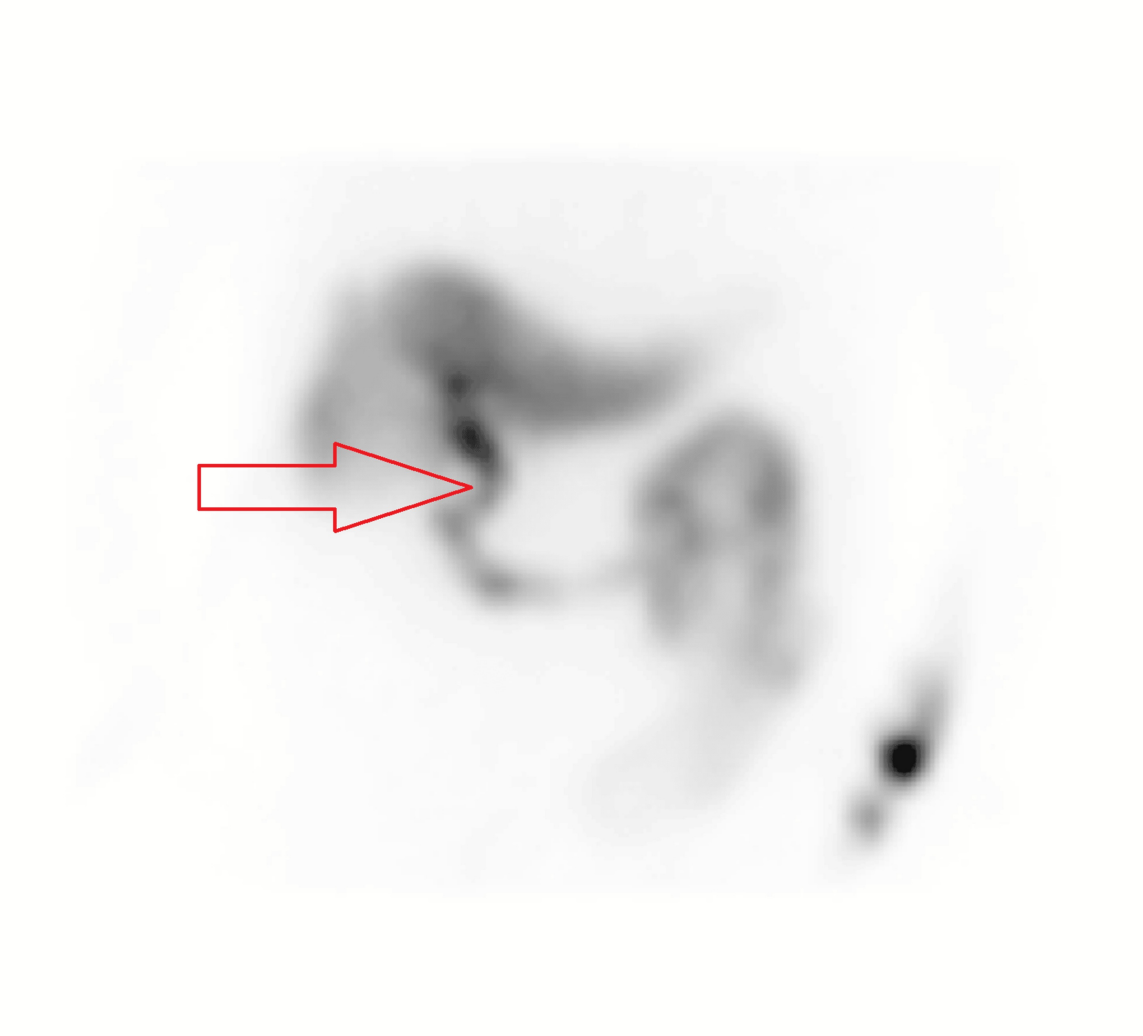
HIDA scan showing contrast flowing freely through biliary tree at the 40-minute time point. HIDA: hepatobiliary iminodiacetic acid. A HIDA image appears blurry naturally.

A hepatobiliary iminodiacetic acid (HIDA) scan (Figure 4) demonstrated prompt uptake of radiopharmaceutical by the liver with normal excretion into the biliary ducts and small bowel. The ejection fraction was 28%, consistent with biliary dyskinesia.

**Figure 5:**
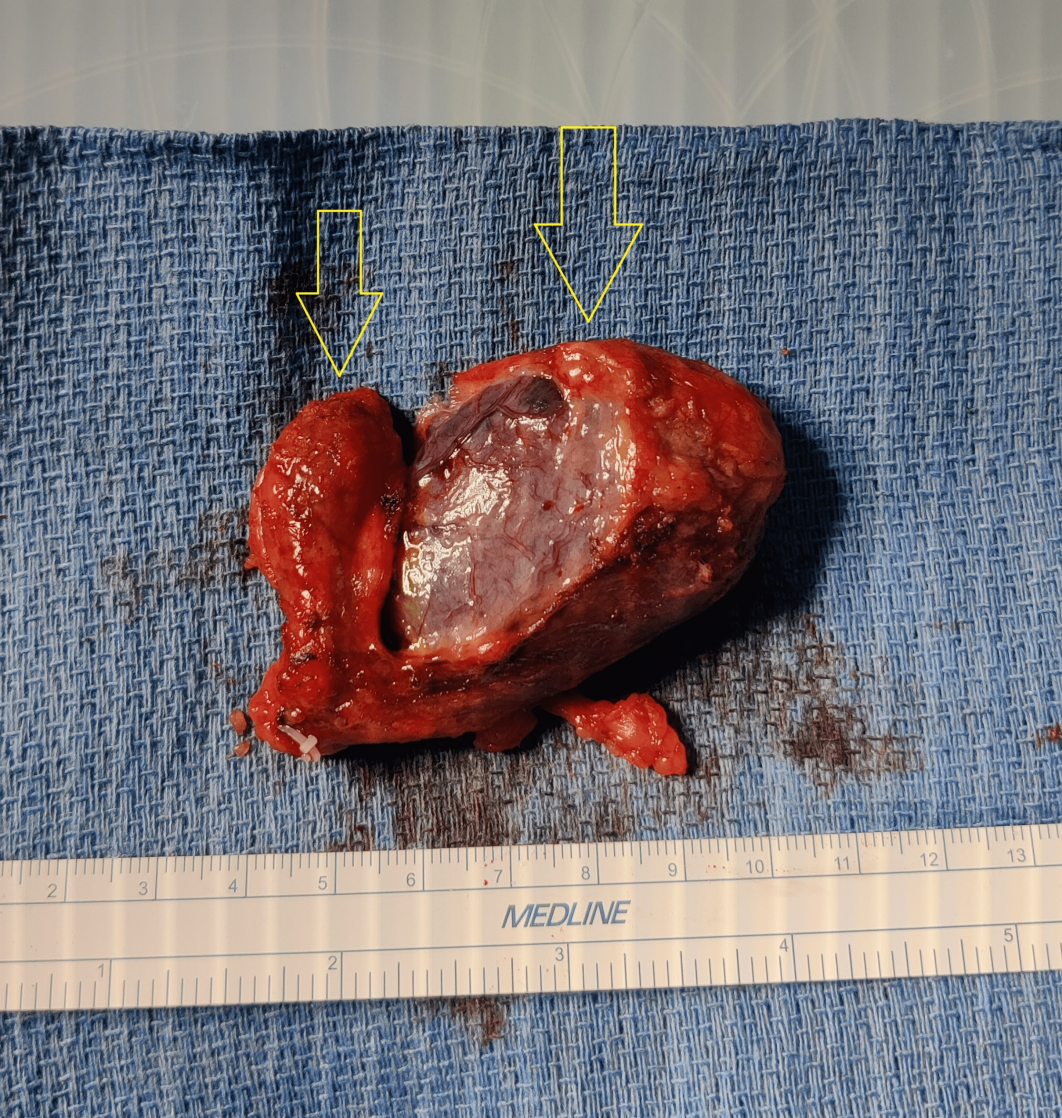
Gross image of duplicated adjoined gallbladder.

The patient was then scheduled for robotic cholecystectomy with the da Vinci Xi and intraoperative indocyanine green (ICG). A 1.25 mg dose of ICG was delivered intravenously 30 minutes before the procedure. A single cystic duct and single cystic artery were first dissected cautiously to ensure the absence of duplicated vessels. The smaller duplicated gallbladder was visualized as attached to the main gallbladder and with no vessel connecting to the main bile duct. However, a small posterior artery was identified which clearly entered the duplicated gallbladder. The critical view of safety was then achieved and the biliary anatomy was confirmed with Firefly and ICG, ensuring conservation of the common bile duct before ligation of the cystic artery and cystic duct. The DG was then meticulously dissected off the liver bed to ensure no extraneous vessels were connected (Figure 5). The patient tolerated the procedure well and went home the same day. He was then seen for follow-up one week later with no adverse effects. 

## Discussion

Preoperative imaging plays a crucial role in diagnosing gallbladder anomalies like DG and determining the presence of associated pathologies such as cholecystitis. Ultrasonography is often the first-line modality due to its widespread availability and non-invasive nature, with reported sensitivities and specificities of 81% and 83%, respectively, for diagnosing acute cholecystitis [3]. Abdominal CT offers higher anatomical detail, with sensitivities of 90% and specificities of 95% for detecting gallbladder inflammation [4]. However, we see in our case that the duplicated gallbladder morphology was missed on CT but later caught on ultrasound, highlighting the importance of utilizing multiple imaging modalities for diagnostics. MRCP, a non-invasive alternative to endoscopic retrograde cholangiopancreatography (ERCP), provides the highest sensitivity (95%) with a smaller specificity (69%) for cholecystitis [5]. While the MRCP’s specificity for cholecystitis might be lower than CT, we still believe that it is an effective and necessary imaging modality in delineating complex anatomy preoperatively, which is supported by the fact that MRCP accurately detected the DG, whereas CT did not.

Robotic-assisted cholecystectomy (RC) was chosen over laparoscopic cholecystectomy (LC) for this case due to its technical advantages, including enhanced precision, superior 3D visualization, and improved instrument articulation [6]. In addition, multiple studies have found RC to have a lower rate of conversion to open cholecystectomy compared to LC [6-8] These features are particularly valuable in complex cases like duplicated gallbladders, where anatomical anomalies increase the risk of complications. While LC remains the gold standard for routine gallbladder removal due to its greater availability and lower cost [6,7], RC offers reduced rates of conversion to open surgery, particularly in challenging or emergent cases [8,9].

The use of indocyanine green (ICG) fluorescence with the Firefly system was pivotal in this case. This technology enhances intraoperative visualization of the biliary anatomy, allowing for the identification of critical structures and reducing the risk of bile duct injury. The preoperative administration of a 1.25 mg dose of ICG provided effective fluorescence with minimal systemic exposure. ICG is typically administered preoperatively to allow sufficient hepatic uptake and biliary excretion and this tailored dosing reflects the need to balance adequate biliary visualization with patient safety, especially in rare and complex cases like DG [2,10,11].

Understanding biliary anatomy is critical in all cholecystectomies, especially in cases involving DG, since there may exist duplicated cystic ducts and/or arteries that supply the extra gallbladder. In fact, over 60 cases of DG with duplicated cystic ducts have been identified in the literature [12]. Duplicated cystic arteries have been noted in literature but not in the DG cases as of yet. To reduce risks such as severing a duplicated cystic duct/artery, the critical view of safety (CVS) is achieved first before ligation. The CVS requires clear identification of the cystic duct and artery, hepatocystic triangle (cystic duct, common hepatic duct, and liver bed), and lastly the exposed cystic plate. The enhanced visualization and precision offered by the robotic platform and ICG fluorescence facilitated the achievement of CVS in this patient, mitigating the risks associated with anomalous anatomy [13]. Fortunately, in this case, the patient’s postoperative course was uneventful, with no biliary or abdominal complications noted at the one-week follow-up.

## Conclusions

Duplicated gallbladders are rare anomalies that increase the risk of complications if unrecognized. This case highlights the importance of multimodal imaging, as CT missed the anomaly while ultrasound detected it, with MRCP being the most sensitive modality for confirming biliary anomalies. Robotic-assisted cholecystectomy was preferred over laparoscopic cholecystectomy due to its superior precision, visualization, and articulation, with indocyanine green fluorescence enhancing biliary visualization and reducing the risk of injury. Achieving the critical view of safety is crucial in complex cases, and the robotic platform improved visualization, aiding safe dissection and minimizing complications. While robotic-assisted cholecystectomy lowers conversion rates to open surgery, its higher cost and operative time should be considered. The patient recovered uneventfully, emphasizing the importance of recognizing a duplicated gallbladder, utilizing comprehensive imaging, and leveraging advanced surgical techniques for optimal outcomes.
